# Dose intense triplet chemotherapy with gemcitabine, carboplatin, paclitaxel with peripheral blood progenitor cell support for six cycles in advanced epithelial ovarian cancer

**DOI:** 10.1038/sj.bjc.6601697

**Published:** 2004-03-02

**Authors:** C Barlow, M Nystrom, C Oesterling, D Fennell, J Ismay, C Gallagher

**Affiliations:** 1 Department of Medical Oncology, St Bartholomew's Hospital, West Smithfield, London EC1A 7BE, UK

**Keywords:** ovarian cancer, gemcitabine, carboplatin, paclitaxel, peripheral blood progenitor cell support

## Abstract

The interval required for haematological reconstitution following myelosuppressive chemotherapy can be reduced by the infusion of autologous peripheral blood progenitor cells (PBPCs). When carboplatin (C) and paclitaxel (P) are followed by granulocyte colony-stimulating factor (GCSF), multiple courses can be given at 10-day intervals with the autologous PBPCs from a unit of whole blood with each cycle. We extended this approach and defined the dose-limiting toxicity and maximum-tolerated dose for the addition of gemcitabine (G) to CP for patients (pts) with EOC in a phase I–II study of increasing doses of G (0, 800, 1000 and 1250 mg m^−2^) over four cohorts with C at area under curve (AUC) 6, plus P at 175 mg m^−2^ 3 h^−1^ every 10 days for six cycles. Granulocyte colony-stimulating factor 5 *μ*g kg^−1^ day^−1^ was given s.c. days 1–10 and 450 ml whole blood was venesected before each treatment, stored untreated at 4°C and reinfused 24 h later. In all, 17 patients with EOC either bulky stage IV or recurrent after treatment-free interval >12 months were treated over 30 months. Of the 17 patients, 13 completed six cycles (one patient stopped early with PD, three with toxicity), interdose interval 9–28 (median 10) days. Delays occurred in four patients due to infection or malaise, and there were no dose reductions. Haematological toxicity was not considered to be dose limiting. Febrile neutropenia was uncommon (2 patients), but grade III/IV thrombocytopenia was seen across all cohorts. Treatment was not delayed for thrombocytopenia and no bleeding complications occurred. Grade III transaminitis was seen in all patients in cohort 4 and grade IV toxicity, considered to be dose limiting, occurred in one. Responses were observed at all dose levels with six CR, seven PR, three SD and one PD. Dose intense GCP was deliverable over six cycles with manageable haematological toxicity, but with dose-limiting hepatic toxicity in cohort 4. The MTD was gemcitabine 1000 mg m^−2^, carboplatin AUC 6, paclitaxel 175 mg m^−2^ given every 10 days for six cycles.

Platinum compounds are the mainstay of treatment in advanced epithelial ovarian cancer. Standard chemotherapy currently comprises a platinum agent in combination with paclitaxel (based on the results of GOG protocol 111 and the Intergroup Trial OV10, although not supported by ICON 3 (McGuire *et al*, 1996; Harper, 1999; Piccart *et al*, 2000)). However, 5-year survival remains 30% or less. It is hoped that improvements in survival can be achieved by the use of newer drugs and employing novel methods of dose intensification such as shortening the interval between treatments, without introducing unacceptable toxicity.

Gemcitabine is a fluorine-substituted pyrimidine antimetabolite, structurally similar to cytosine arabinoside, which exhibits widespread antitumour effects both *in vitro* and *in vivo*. It becomes incorporated into DNA, leading to DNA strand breaks (after addition of a subsequent nucleotide) and also disturbance of DNA repair mechanisms (Huang *et al*, 1991; Plunkett *et al*, 1996). Studies suggest a synergism between gemcitabine and platinum analogues (Bergman *et al*, 1996; van Moorsel *et al*, 1999), which is thought to be due to gemcitabine's effects on DNA repair and its different toxicity profile (Aapro *et al*, 1998), thereby lending itself to combination therapy. Phase I/II trials have shown that gemcitabine has activity in advanced EOC in both chemo-naive patients (Underhill *et al*, 2001) and those with disease resistant to platinum and paclitaxel (Lund *et al*, 1994; Shapiro *et al*, 1996; Friedlander *et al*, 1998). The combination of gemcitabine with platinum and paclitaxel has already been reported to produce high response rates in EOC, but with dose-limiting haematological toxicity (Geertsen *et al*, 1999; Hansen *et al*, 1999a, 1999b; Poole *et al*, 2001; Hansen, 2002).

The experience of dose intensification with conventional marrow ablative high-dose therapy, often with single agents (Ledermann *et al*, 2001), has not proven to be beneficial for most patients. An alternative approach is to use multiple agents, with known activity, at full dose, but with a short intercycle time interval to prevent tumour recovery between treatments. The use of granulocyte colony-stimulating factor (GCSF) alone has not been sufficient to increase the dose intensity of standard multicycle chemotherapy; however, when combined with the collection and reinfusion of peripheral blood progenitor cells (PBPC), there can be a doubling of dose intensity for carboplatin and paclitaxel (Pettengell *et al*, 1995, 1999; Woll *et al*, 1995, 2001).

This study was designed to investigate whether treatment of advanced EOC, with gemcitabine, carboplatin and paclitaxel given every 10 days for six cycles, can be tolerated with the support of GCSF and PBPC from autologous transfusion. The primary objectives were to define the maximum-tolerated dose (MTD) and dose-limiting toxicities (DLT) of this combination and schedule, using fixed doses of carboplatin and paclitaxel, with escalation of the dose of gemcitabine and secondarily to evaluate the response rate and duration of response following this treatment.

## MATERIALS AND METHODS

### Patient characteristics

In all, 17 patients were enrolled into the study between February 2000 and June 2002. All had histologically proven EOC, were aged 18–65 years, performance status 0–2, with a life expectancy of >3 months. Other eligibility criteria were measurable or evaluable disease by RECIST criteria, normal haematological function, adequate renal function (GFR >60 ml min^−1^) and hepatic function (LFTs <2 × ULN). Patients were either previously untreated with stage IV or bulky unresectable stage III disease (10), or had relapsed with a treatment-free interval of >1 year (7) (see Table 1

**Table 1:** Patient characteristics

Number	17
Age (years), median	27–73 (56)
*Histology*	
Serous	10
Adenocarcinoma	6
Clear cell	1
	
*Stage*	
3	
Biopsy only	1
Suboptimal debulk	4
Optimal debulk	2
4	
Biopsy only	2
Suboptimal debulk	1
Recurrent	7

).

Patients were excluded if there was a concurrent malignancy (other than nonmelanoma skin cancer), central nervous system metastases, presence of atypical red blood cell antibodies, hepatitis B surface antigen or anti-hepatitis C antibodies, HIV 1 and 2 antibodies, a previous adverse reaction to carboplatin or paclitaxel or contraindication to the use of platinum. The protocol was reviewed by the institutional research ethics committee and all patients gave written informed consent. The support for GCSF costs was received from Amgen UK.

The pretreatment evaluation included a full history and physical examination with all lesions measured by physical examination confirmed by a second physician. Laboratory investigation included full blood count, biochemical profile (to assess liver and renal function) and assay of CA 125. Diagnostic imaging of measurable lesions was by chest X-ray, computed tomography (CT) or magnetic resonance imaging.

### Study design

Treatment was given in five cohorts each comprising three patients. Chemotherapy was administered, according to cohort, on day 1 of a 10-day cycle, for six cycles. GCSF (5 *μ*g kg^−1^) was given subcutaneously on days 2–9. On day 10, 750 ml of blood was venesected immediately prior to the next cycle of chemotherapy and stored unprocessed at 4°C (according to standard guidelines proposed by the British Committee for Standards in Haematology Blood transfusion Task Force; Voak *et al*, 1993). The autologous blood was reinfused at least 18 h postchemotherapy on cycles 2–6 prior to recommencing GCSF (see Table 2

**Table 2:** GCPq10 chemotherapy regimen by cohort

	**Cohort 1**	**Cohort 2**	**Cohort 3**	**Cohort 4**
Carboplatin day 1	AUC 6	AUC 6	AUC 6	AUC 6
Paclitaxel day 1 (mg m^−2^)	175	175	175	175
Gemcitabine day 1 (mg m^−2^)	—	800	1000	1200

AUC=area under curve.

and Figure 1

**Figure 1:**
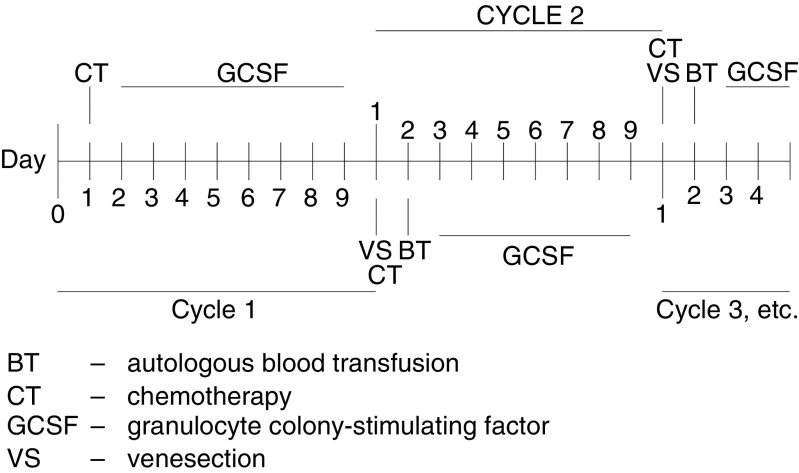
Treatment overview.

). No prophylactic antibiotics were given. Intravenous antibiotics were given for febrile neutropenia and blood product support was given according to unit policy.

### Dose escalation

The dose of gemcitabine was escalated through cohorts 1–4 according to Table 2. At each dose level, the first patient completed the full cycle of treatment before the remaining patients were entered in case of unexpected toxicity. Each cohort completed treatment before escalation to the next dose level. The dose was escalated until the MTD was reached. As haematological toxicity was expected, this was defined as the dose at which two of three patients experienced nonhaematological DLT. This was defined as grade III or IV toxicity, according to National Cancer Institute common toxicity criteria. If two of the three patients in a cohort experienced DLT, a further three patients were recruited at the same dose and MTD was established if DLT occurred in another patient.

### Response assessment

Computed tomography scans to assess response were carried out after four cycles and 4 weeks after the sixth cycle. Treatment was continued to six cycles provided there was no evidence of progressive disease. CA 125 assays were also carried out at the start of each cycle. Follow-up after completion of treatment was monthly with physical examination and CA125, with radiological investigations as indicated by these findings.

### Statistical analysis

Means, medians and proportions were calculated as appropriate. This is a descriptive study and no intergroup comparisons were made.

## RESULTS

In all, 17 patients were recruited into the study. Dose escalation of gemcitabine was achieved up to 1250 mg m^−2^, together with carboplatin area under curve (AUC) 6 and paclitaxel 175 mg m^−2^ every 10 days, for six cycles.

All six cycles of treatment were completed by 13 of the 17 patients (Table 3

**Table 3:** Treatment delivery

**Cohort**	**Cycle no. (mean)**	**Cycle length – days (mean)**	**Delayed cycles (no.)**	**Dose reductions (no.)**
1	6	11.4	2	0
2	6	11.3	2	0
3	6	10.3	2	1
4	5	10.0	0	0
3 repeat	4.2	10.0	1	0

). One patient discontinued treatment after four cycles due to progressive disease and three patients stopped treatment early due to toxicity. All are included in the analysis on an intention-to-treat basis.

The average cycle length was 10.5 days (range 9–28 days). Standard dose intensity was taken as carboplatin AUC 6 q21 days, paclitaxel 175 mg m^−2^ q21 days and gemcitabine 1250 mg m^−2^ day 1 and 8 q21 days. In comparison, we achieved double the dose intensity of carboplatin AUC 5.04 week^−1^ and double the dose intensity of paclitaxel 147 mg m^−2^ week^−1^, but only 1.01 times the dose intensity of gemcitabine at 840 mg m^−2^ week^−1^ Dose reductions, due to nonhaematological toxicity, were necessary for two patients. Four patients had delays due to infectious complications or malaise, resulting in a total of six of the 93 (6%) delayed cycles (Table 3).

Haematological toxicity was expected, detected either by day 10 blood count or by investigation of symptoms between treatment cycles (Table 4

**Table 4:** Grade III/IV haematological toxicity

		**Grade III/IV neutropenia**			**Grade III/IV thrombocyctopenia**		
**Cohort**	**Total no. of cycles**	**No. cycles**	**%**	**Nadir**	**No. cycles**	**%**	**Nadir**
1	18	3	17%	0.0	3	17%	28
				0.8			20
				N/A			13
							
2	18	2	11%	0.1	3	17%	38
				0.1			28
							12
							
3	18	0			7	39%	15
							21
							7
							8
							18
							45
							5
							
4	21	0			3	14%	25
							37
							8
							
3 repeat	18	1	6%	0.1	4	22%	29
							33
							7
							9
							
Total	93	6	6%		20	22%	

N/A=not available.

).

Grade III or IV thrombocytopenia was the most prevalent toxicity occurring in 20 of the 93 (22%) cycles. The median platelet counts and ranges on day 10 were 214 (20–281), 106 (28–281), 71 (15–119), 70 (25–206) and 90 (7–306) for cohorts 1, 2, 3, 4 and 3 repeat, respectively.

Platelet transfusions were given for counts below 10. Treatment was repeated on day 10 regardless of the platelet count and no cumulative toxicity or bleeding complications occurred.

Grade III or IV neutropenia occurred in a total of five of 93 (5%) cycles. The median day 10 neutrophil counts and ranges for cohorts 1, 2, 3, 4 and 3 repeat were 10.2 (0.8–30.9), 9.5 (1.1–59.6), 17.5 (5.2–59.4) 18.8 (9.6–39.9) and 10.0 (3.6–52.3). Two patients required admission with neutropenic sepsis. The first patient to develop febrile neutropenia received treatment in cohort 2. As the episode was uncomplicated, this was not considered to be a DLT. The second patient discontinued treatment as a result of febrile neutropenia after cycle 3 in the final cohort (cohort 3 repeat).

Nonhaematological toxicity is described in Tables 5

**Table 5:** Other grade III/IV nonhaematological toxicity

Cohort	**Total no. of cycles given**	**Infection cycles (%)**		**GI cycles (%)**		**Malaise cycles (%)**		**Dyspnoea cycles (%)**	
1	18	1	(6%)	0	(0%)	2	(11%)	0	(0%)
2	18	0	(0%)	0	(0%)	0	(0%)	4	(22%)
3	18	0	(0%)	4	(22%)	4	(22%)	4	(22%)
4	21	0	(0%)	1	(5%)	2	(10%)	0	(0%)
3 repeat	18	1	(6%)	0	(0%)	3	(17%)	0	(0%)
									
Total	93	2	(2%)	5	(5%)	11	(12%)	8	(9%)

and 6

**Table 6:** Hepatic toxicity – all grades

**Cohort**	**Total no. of cycles**	**Toxicity no. of cycles (%)**		**Grade**
1	18	0	0%	
2	18	3	17%	I/II
3	18	5	28%	I/II
4	21	7	33%	I/II
		5	24%	III/IV
3 repeat	18	2	11%	I/II
				
Total	93	22	24%	

, with grade III malaise in 10 cycles, grade III GI toxicity in five cycles and grade III dyspnoea in eight cycles (due to one patient developing pneumonia and one patient having a pulmonary embolus, that is, not directly due to drug-related pulmonary toxicity). One patient developed a rash and another had an allergic reaction to carboplatin, requiring adjustment in premedication and a slower rate of infusion in subsequent cycles. No renal toxicity was observed at any dose level.

Liver function abnormalities appeared related to the dose of gemcitabine and were not seen in cohort 1 (Table 6). One patient in each of cohorts 2 and 3 had a rise in alanine transaminase (ALT), gamma glutamyl transpeptidase or alkaline phosphatase (total of eight cycles) and all four patients in cohort 4 developed abnormalities. Grade III toxicity was seen in four cycles (three patients). Grade IV toxicity was seen in one patient, with >20 times upper limit of normal rise in ALT for 2 days, returning to normal over 2 weeks, who also became jaundiced with a grade I rise in bilirubin. Her hepatitis serology remained negative and liver biopsy revealed nonspecific changes consistent with a drug-induced hepatitis. Although the formal definition of DLT had not been reached, it was decided, in view of the trend of increasing liver toxicity, to return to cohort 3 for a further study. In the repeated cohort 3, one patient experienced grade I or II hepatic toxicity (two cycles), as seen previously.

All patients were evaluable for response (Table 7

**Table 7:** Outcome

**Cohort**	**CR**	**PR**	**SD**	**PD**
1	1	2	—	—
2	—	2	1	—
3	2	1	—	—
4	2	1	—	1
3 repeat	1	1	2	—

Total: six CR, seven PR, three SD, one PD, overall response rate 13 of the 17 – 76%.

CR=complete response, PR=partial response, SD=stable disease, PD= progressive disease, by RECIST criteria.

). Responses were seen at all dose levels. Complete response was achieved in six patients (35%) and a partial response in seven (41%). Three patients had stable disease and progressive disease was seen in one. The overall response rate was 76% (95% CI: 56–96%).

One patient died prior to completing treatment due to bowel obstruction and postoperative complications with a normal full blood count.

The median length of follow-up is now 20 (range 2.9–34.0) months, with a progression-free survival of 11 (range 1.3–28.8) months.

## DISCUSSION

The results of this study demonstrate that it is feasible to combine gemcitabine with carboplatin and paclitaxel and to dose intensify by delivering treatment every 10 days, with appropriate haematological support to achieve a dose intensification of 2.1 times conventional carboplatin and paclitaxel doses, but only 1.01 times the gemcitabine dose intensity because of dose-limiting hepatic toxicity.

Ovarian cancer is a chemosensitive condition and has therefore, as with other chemosensitive tumours, generated considerable interest in increasing cytotoxic dose intensity, with the aim of curing more patients. To date, the experience with conventional high-dose therapy in this disease has not been encouraging. In particular, it is clear that patients with bulky disease do not benefit from this form of treatment and only the subgroup of patients in complete remission at the time of high-dose therapy has been shown to have a survival advantage over those whose transplants were carried out in partial remission or less (Ledermann *et al*, 2001). There may be a number of possible explanations for this. Studies have often used single-agent cytotoxics, which have not had a particularly high activity in epithelial ovarian cancer. Also, even with bone marrow support, the recovery time following a single marrow ablative therapy is necessarily lengthy, allowing for tumour recovery during this period.

It may be that alternative models of dose intensification, using combinations of more active agents, over short time intervals, may be more effective and we have shown one such strategy to be possible.

The combination of gemcitabine, carboplatin and paclitaxel has been associated with high response rates, both in our study of patients with bulky or recurrent disease and previous reports (Geertsen *et al*, 1999; Hansen *et al*, 1999a, 1999b; Poole *et al*, 2001; Hansen, 2002). With conventional 3-weekly scheduling, however, haematological toxicity is dose limiting. In order to dose intensify, we have used this three-drug combination every 10 days, with GCSF-driven haematopoietic stem cell support from whole blood. Previous reports have shown a modest increase in dose intensity (1.34) to be possible with the use of GCSF alone (Woll *et al*, 1995). However, a doubling of dose intensity may be achieved by collection and reinfusion of haematopoietic stem cells (Pettengell *et al*, 1995). These can be collected in whole blood after GCSF priming by venesection immediately prior to chemotherapy. They may be stored, unprocessed at 4°C for 48 h prior to reinfusion, allowing for a practical and straightforward procedure that does not necessarily require an overnight hospital admission. The optimal collection occurs around days 10–12 following carboplatin and paclitaxel (Pettengell *et al*, 1999), hence the rationale for our intercycle interval.

The incidence of febrile neutropenia was low at two of the 93 cycles and was not found to be dose dependent. The most common toxicity was thrombocytopenia, which was seen across all cohorts. However, following the experience of Pettengel *et al*, we did not delay treatment for thrombocytopenia and saw no cumulative toxicity, even though patients were treated with a platelet count as low as 7 at the time of chemotherapy.

We had anticipated that bone marrow toxicity would be common with this regimen, but found that with PBSC support we were not restricted by what would be considered to be conventional dose-limiting haematological toxicity. In addition, patients who have subsequently relapsed have been able to receive further cytotoxic chemotherapy without bone marrow compromise.

It appears from our study that the dose of gemcitabine, in combination with platinum and paclitaxel, cannot be escalated above 1000 mg m^−2^ every 10 days, owing to hepatic toxicity. Phase I studies have suggested that gemcitabine, scheduled weekly for 3 weeks out of every 4, provides optimal activity (Allerheiligen *et al*, 1994; Fossella *et al*, 1997). In terms of toxicity, most data are available with gemcitabine as a single agent, given according to the above schedule in doses ranging from 800 to 1250 mg m^−2^. Transient elevations in serum transaminases are well described, occurring in approximately half of all patients; however, grade III/IV toxicity is uncommon, occurring in <10% of patients. There have been two case reports in the literature of mortality from liver failure attributed to gemcitabine administration after other causes were ruled out (Dobbie *et al*, 1998; Coeman *et al*, 2000). Although most reports suggest transient toxicity, unrelated to the total cumulative dose or duration of exposure to gemcitabine, both fatalities occurred in patients who had undergone treatment for at least 15 weeks. Our own observations certainly support a dose effect, with grade III/IV toxicity only occurring at doses >1000 mg m^−2^. It is difficult to extrapolate these data entirely, as we do not know the influence of the other drugs and our different schedule on hepatic toxicity. It is likely that higher doses cannot be tolerated at 10-day intervals for protracted periods.

In conclusion, while dose intensification was possible with paclitaxel and carboplatin, we were not able to increase the dose intensity of gemcitabine due to hepatic toxicity. The thrombocytopenia was manageable with PBSC support, but makes this regimen unlikely to be applied outside the research setting because of the risk of bleeding, particularly if combined with other predisposing factors. However, early intensification has been shown to be important in other tumour types and perhaps with the advent of potential maintenance therapies in ovarian cancer, such as weekly taxol, angiogenesis inhibitors and signal transduction modulators, dose intense multiagent treatment could be considered to provide remission induction prior to maintenance, analogous to the treatment of acute leukaemia.
